# A luminescent Zn-MOF exhibiting high water stability: selective detection of Cr(VI) ion and treatment activity on sepsis

**DOI:** 10.1080/15685551.2021.1953239

**Published:** 2021-07-27

**Authors:** Ying Wan, Xu-Mei Chen, Qian Zhang, Hong-Biao Jiang, Ran Feng

**Affiliations:** a Emergency Department, Affiliated Hospital of Shandong University of Traditional Chinese Medicine, Jinan, Shandong, China; b Department of Classical Chinese Medicine, Affiliated Hospital of Shandong University of Traditional Chinese Medicine, Jinan, Shandong, China

**Keywords:** MOF, Cr(VI) ion detection, sepsis treatment, inflammatory factor storm

## Abstract

A fresh metal-organic framework (MOF) based on the Zn ions as the metal ions has been prepared via the solvothermal method, and its chemical formula is [Zn(byia)(DMF)]·1.5DMF·7H_2_O (**1**, byia =  5-(benzimidazol-2-yl) isophthalic acid). It is worth noting that the compound **1** has excellent water stability (which can be maintained in the water for at least a month). Most fascinating, in water, the compound **1** exhibits the strong blue luminescence, which can only be selectively quenched via the contaminant of the Cr_2_O_7_^2–^ ion. The selective luminescence quenching with low limits of detection and high values of *K*_sv_ proved its better sensing property, which can be compared with the contemporary materials. To development new strategy for the sepsis treatment, the biological activity and mechanism of the compound was explored. Firstly, the ELIA detection was performed in this experiment to assess the inhibition of compound against inflammatory factor storm during sepsis. Then, the inflammatory response in the immune cells was assessed by real time RT-PCR.

## Introduction

Due to the high mortality rate and huge medical expenses of sepsis, it has attracted more and more attention from clinicians. According to statistics, a total of 3 million patients in the United States have developed sepsis in 2019, with a mortality rate exceeding 200,000, and the incidence rate will increase in the further [1,2]. The increased incidence of sepsis is believed to be related to the aging of the population and the improvement of modern scientific diagnostic techniques [3]. During sepsis, there was usually combined with an inflammatory factor storm, showed as the increased level of inflammatory cytokines releasing, which serious disturbed the clinic treatment.

In the past few years, the metal-organic frameworks (MOFs) have been extensively investigated because of their excellent and versatile applications in the fields of ferroelectricity, proton conduction, luminescence, biomedicine and magnetism as well as some other fields [4–7]. The luminous MOFs can produce the photoluminescence and in-depth serve as the materials of luminescent sensing. They have been widely studied in some toxic pollutants sensing, for instance, organic small molecules and inorganic heavy metal ions, and they have a significant function in human health and environmental protection [8–12]. The reasonable design and the establishment of the MOFs have aroused great interest. So far, many fascinating MOFs have been designed and then synthesized through using the mold design and utilizing the methods of crystal engineering. Nevertheless, the acquisition of the target MOF contains ideal performances and pre-designed structure remains a huge challenge. Many factors, for instance, the metal centres coordination geometry, the value of pH, the architecture for organic ligands, temperature and the ratios of metal–ligand, these factors may influence the process of synthesis and result in the ultimate structural diversities [13–17]. In these above factors, organic connectors with appropriate metal centers play an important role in the detection of the performances and structure of the MOFs. Therefore, the design and reasonable choice for organic ligands are the key to the establishment of MOFs [18,19]. We choose the 5-(benzimidazol-2-yl) isophthalic acid (H_2_byia), a kind of rigid polyaromatic ligand, as an organic connector to establish stable MOFs according to following two factors: One is that the typical metal carboxylate structure can generate various structures; the other is that the polyaromatic ligand can promote the generation of π-π interactions and enhance the stability of the structure. In this research, a new metal-organic framework (MOF) based on the Zn impeller has been prepared via the solvothermal method, and its chemical formula is [Zn(byia)(DMF)]·1.5DMF·7H_2_O (**1**, byia =  5-(benzimidazol-2-yl) isophthalic acid). It is worth noting that the compound **1** has excellent water stability (which can be maintained in the water for at least a month). Most fascinating, in water, the compound **1** exhibits the strong blue luminescence, which can only be selectively quenched via the contaminant of the Cr_2_O_7_^2–^ ion. The selective luminescence quenching with low limits of detection and High valueso of *K*_sv_ proved its better sensing property, which can be compared with the contemporary materials. Through serious of biological experiments, the treatment effect of compound against the sepsis was evaluated, and at the same time, the detailed mechanism was investigated. The ELIA detection results suggested that compound could obviously inhibited the generation of the inflammatory factor storm during sepsis with the dose-dependent pattern. Furthermore, real time RT-PCR revealed that the inflammatory response in the immune cells was also suppressed by the compound exposure dose dependently.

## Experimental

### Chemicals and measurements

The materials utilized in this research were acquired from market and then they were utilized without additional purification unless otherwise specified. With the X-ray powder diffractometer of Bruker D8 ADVANCE, we can harvest the date of the powder X-ray diffraction (PXRD). We commonly can utilizing the FT-IR spectrometer of EQUINOX-55 to record the FT-IR spectra from 4000 cm^−1^ to 400 cm^−1^. And through utilizing the Elemental analyzer of Perkin-Elmer 2400 C to analyze the element. For the determinations of thermogravimetry (TGA), we can conduct that through utilizing the equipment of Netzsch TG209F3 with 10°C·min^−1^ heating rate in atmosphere of N_2_. The determinations of solid photoluminescence were recorded by the Horiba fluorescence contains the solid sample frame. The fluorescent spectra in the solution were recorded on a FluoroMax-4 fluorescent spectrophotometer.

### Preparation and characterization for [Zn(byia)(DMF)]·1.5DMF·7H_2_O (1)

Through 0.01 mmol and 0.003 g H_2_byia, 0.02 mmol and 0.006 g Zn(NO_3_)_2_ · 6H_2_O, 1.0 mL of N,N-dimethylformamide (DMF) and 1.0 mL on H_2_O mixed with 0.1 mL 1,4-Diazabicyclo [2.2.2] octane (DABCO, 2.0 g dissolved into the DMF of 10 mL), and 0.075 mL of HNO_3_ (2.2 mL dissolved into the DMF of 10 mL), we can acquire a mixture, and then the obtained mixture was stored into the 20 mL capped bottle, after that heated it for two days at 85°C. Then, after the mixture cooling to the ambient temperature, we acquired the colorless crystals with rod-like. The crystals were harvested, and cleaned by using the H_2_O and then dried in the air (with 68% yield, on the basis of H_2_byia). Elemental analysis (%) calcd. for compound **1**: N, 9.66, H, 6.05 and C, 41.38; found: N, 8.94, H, 5.87 and C, 42.07. IR (KBr, cm^–1^): 450 (m), 559 (w), 683 (m), 731 (s), 785 (s), 987 (m), 1104 (m), 1256 (m), 1384 (s), 1580 (s), 1660 (s), 1704 (s), 2936 (m), 3415 (s).

We can analyze the data of strength through software of crysalispro, after that transformed the intensity data into the files of HKL. We utilized diffractometer of Oxford XcaliburE to get the data of X-ray. Complex **1**’s original skeleton model was built through utilizing SHELXS based on the direct manner, and then modified the model through to SHELXL-2014 according to least square manner. Anisotropic parameters were mixed with complex **1**’s atoms of non-hydrogen. Afterwards, the entire hydrogen atoms are geometrically fixed to C atom they are linked to through utilizing the command of AFIX. The refinement along with the crystallographic parameters for the complex **1** are described in detail in Table 1.

**Table 1.** The parameters of crystallography and the refinement for the complex **1.**

Empirical formula	C_15_H_10_N_2_O_5_Zn
Formula weight	363.62
Temperature/K	298.15
Crystal system	Trigonal
Space group	R-3
a/Å	21.236(3)
b/Å	21.236(3)
c/Å	22.3396(14)
α/°	90
β/°	90
γ/°	120
Volume/Å^3^	8725(3)
Z	18
ρ_calc_g/cm^3^	1.246
μ/mm^−1^	1.287
Data/restraints/parameters	4187/0/209
Goodness-of-fit on F^2^	1.035
Final R indexes [I ≥ 2σ (I)]	R_1_ = 0.0381, ωR_2_ = 0.0938
Final R indexes [all data]	R_1_ = 0.0586, ωR_2_ = 0.1019
Largest diff. peak/hole/e Å^−3^	0.39/-0.40
CCDC	2,010,752

### IL-18 and TNF-α measurement

The ELISA detection was carried in this present research to determine the TNF-α and IL-18 releasing levels in the sepsis mice after treated with compound. This conduction was accomplished under instructions’ guidance with slight modification, and the experiments of animal were all authorized via the Animal Ethics Committee of Chinese Medicine Nanjing University (Nanjing, China). In this research, 50 BALB/c of the mice were acquired from the Laboratory Animal Center of Chinese Medicine Nanjing University (Nanjing, China), and then cultured at a standard environment of 20–25°C. Then, we injected the LPS into mice at 5 mg/kg concentration to construct the model of sepsis. After that, the compound was utilized to conduct the treatment at 1 mg/kg, 2 mg/kg and 10 mg/kg. Finally, the abdominal suspension was collected and then the content of the inflammatory cytokines was measured.

### Nf-κb *and* p53 *determination*

To determine the *p53* and *nf-κb* relative expression in immune cells, the real time RT-PCR method was implemented in this current research, which was strictly in accordance to the instructions of the manufactures’ protocols. In brief, the BALB/c mice were injected with LPS at 5 mg/kg concentration to construct the model of sepsis. After that, the compound was utilized to conduct the treatment at 1 mg/kg, 2 mg/kg and 10 mg/kg. Then, the immune cells in the abdominal suspension was collected, and we extracted the overall RNA via the TRIZOL reagent (Sigma, St. Louis, MO, USA) on the basis of the instructions of manufacturer. The instrument of NanoDrop 2000 C was applicated to detect the quality and concentration. Afterward, the cDNA was synthesized and the *p53* and *nf-κb* relative expression was detected through utilizing the SYBR Green Master Mix (Roche). All of the results were standardized through utilizing the 2^−ΔΔCt^ approach.

## Results and discussion

### Molecular structure

**Figure 1.**
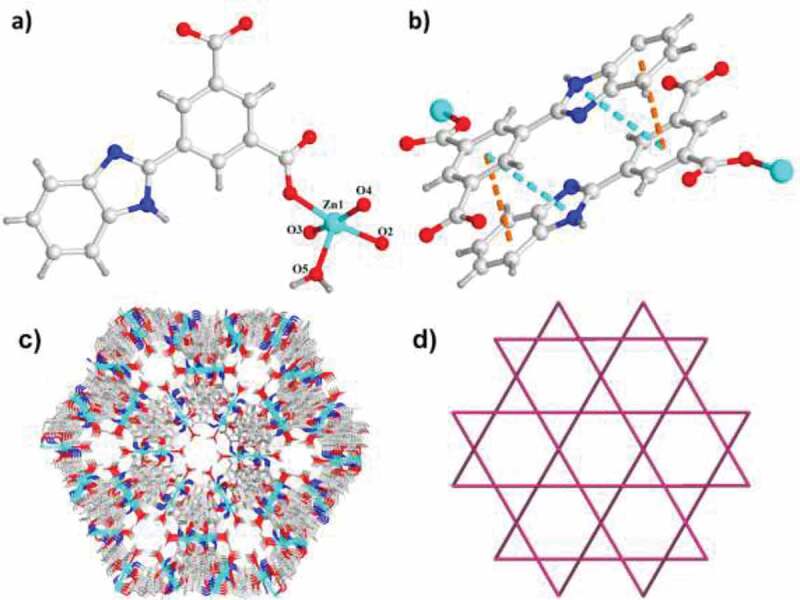
(a) Complex **1**’s asymmetry unit view. (b) The interactions of π–π between the neighbouring ligands. (c) Complex **1**’s three-dimensional skeleton displaying the one-dimensional channels. (d) Complex **1**’s 4-linked topology ***nbo***

The single crystal diffraction exhibits that the compound **1** is crystallized in the hexagonal space and the space group of *R-*3. As expected, the two ligands triumphantly generated a interlaced π–π stacking in complex **1**’s three-dimensional structure. As illustrated in the Figure. 1(a), complex **1**’s asymmetry unit contains a Zn(II) ion, a completely deprotonated ligand of byia^2-^ and a coordinated molecule of water. The Zn(II) ion is pentacoordinated, which completed by five distinct oxygen atoms in the ligands of byia^2-^, generating a geometry of trigonal bipyramidal. All of ligands are arranged in parallel and tell lateral shift is about 1.1 Å. Two typical interactions of π–π are generated by benzimidazole group form the other ligand and benzene ring from each isophthalic acid, and the distances from face to center respectively are 3.5 and 3.6 Å (Figure 1(b)). The classical Zn impeller SBUs are connected via four 2-linked ligands of byia and generate a three-dimensional porous skeleton (Figure 1(c)). In structure, six zinc paddles and eighteen ligands generate a special cage. Obviously, 12 ligands generate six pairs of the interactions of the π-π in each of the cage, producing two small relatively windows, making the cage relatively absolute of the entire structure. Compound **1** exhibits a channel of 3.52 × 3.52 Å through the direction of axis c. The total volume of solvent-accessible each unit cell calculated through the PLATON is 3491 Å^3^, accounting for about 40.8% of the volume of cell. In general, the SBU of Zn impeller is considered as the 4-linked square node, the entire structure can be expressed as the topology of ***nbo***, and composed of a kind of [6^8^] tiles (Figure 1(d)).

### PXRD measurements and TGA profiles

**Figure 2.**
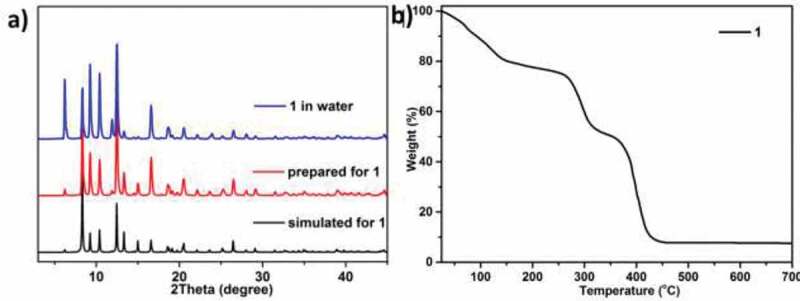
(a) The diagram of PXRD for complex **1**. (b) Complex **1**’s curve of TGA curve

At the aim of detecting the products’ phase purity, the experiments for the PXRD were conducted for the complex **1** utilizing its fresh synthesized samples (Figure 2(a)). The peak positions of PXRD diagrams of the simulation and experiment are in line with each other, this reflects that for the products of crystal with bulk-shape, crystal architectures are a real representative. The differences for the strength can attribute to preference. MOFs’ water stabilities are the significant aspects in their practical applications. To study this compound‘s hydrolytic stability, the samples of several milligrams were soaked into the water for a month and the diagram of PXRD were harvested when the sample was dried. The diagrams of PXRD clearly revealed that in the water, the crystallinity of this compound was maintained, and there was small change of strength in the diagram, this could be attributed to the skeleton shrinkage caused by solvent effect. Complex **1**’s outstanding water stability might because of introducing the interactions of π–π, and another significant reason might be the hydrophobic small cavity generated in the compound **1** prevents the water from entering the unstable zinc nodes. Thermogravimetry (TGA) is utilized to research complex **1**’s thermal stability in the atmosphere of nitrogen with at 5°C·min^–1^ heating rate at the temperature range of 25°C–800°C, as illustrated in the Figure 2(b), the first weightlessness is under 149°C, which pertains to the wiping of the guest molecules of H_2_O (with the calculated value of 20.1%). In the temperature range of 149°C–313°C, the weightlessness pertains to the removal of terminal coordinated molecules of DMF (with the calculated value of 26.3%). The decomposition of ligands is conduct at 313°C (the calculated value is 45.9%) and the power of ZnO is kept at 7.7% at 439°C.

### Photoluminescent sensing properties

**Figure 3.**
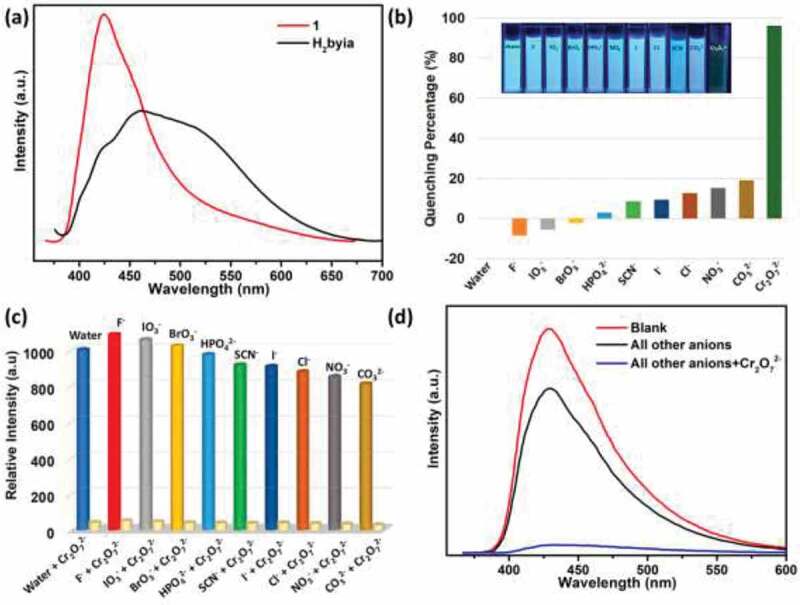
(a) The solid photoluminescence spectrum for complex **1** and ligand; (b) the efficiency of luminescence quenching after introducing diverse anions to complex **1**’s dispersion in the water. (c) The luminescence strengths of complex **1** soaked into the mixed anions with Cr_2_O_7_^2 –^ ions (1 mM) and 1 mM pure separate anions. (d) The MOF photoluminescence spectra in this blank water solution, in the existence of the water solution of some other anions (1 mM) (I^−^, Cl^−^, F^−^, HPO_4_^2-^, BrO_3_^−^, IO_3_^−^, NO_3_^−^, SCN^−^ and CO_3_^2-^) and after adding 1 mM of the Cr_2_O_7_^2-^ ions

Complex **1**’s solid photoluminescence spectra harvested under the amount temperature reveal 424 nm emission peak under 350 nm excitation, this chiefly equivalent to the highly H_2_ byia ligand of π-delocalized planar (Figure 3). Fluorescent emission is caused by the transition of π → π* between pure ligand π*-antibonding orbital and π-bonding orbital, and this transition is emitted at 460 nm under 365 nm excitation. It is obvious that complex **1**’s fluorescence emission is originated from the ligand of H_2_byia because of Zn(II) ions configuration of d^10^. The emission blue shift of complex **1** relative to the blue shift of free ligand. This may appear in the restrictive zinc(II) coordination structure with ligand of H_2_byia and dicarboxylic acid, resulting in the ligand-to-ligand charge transfer (LLCT) over short distance of the neighbouring ligands. Because weak interaction between the neighboring ligands is improved, the emission intensity is obviously enhanced.

At the aim of researching complex **1**’s sensing performances, we added the raw water solution for that diverse KX anions (X = I^–^, Cl^–^, F^–^, HPO_4_^2–^, BrO_3_^–^, and IO_3_^–^, NO_3_^–^, SCN^–^, CO_3_^2–^, as well as Cr_2_O_7_^2–^) into Zn–MOF water suspension to remain the anions concentration of 1 mM. Various anions have diverse effects against the luminescence. BrO_3_^–^, IO_3_^–^, F ^–^ have little increase on the strength of fluorescence, whereas other anions have slight quenching effect on fluorescence strength. Fascinatingly, we found the most obvious quenching effects under the condition of the Cr_2_O_7_^2 –^ ion (Figure 3(a)). Only with the existence of the chromate ions, the fluorescence strength nearly extinguished fully. The efficiency of quenching for other anions was extremely low, but the efficiency of quenching is increased to 96% after adding the Cr_2_O_7_^2 –^ ion (Figure 3(b)). The selective quenching of the chromate ions was investigated through other anions interference experiments. The existence of other anions has no obvious quenching effect on the dispersion of MOF, whereas, after adding the Cr_2_O_7_^2 –^ ions, the quenching effect of MOF nearly disappears (Figure 3(c)). Therefore, the selective sensing for the chromate ions is independent of the existence of some other ions in the water solution. When all of other anions (1 mM) are exist in the water, the luminescent strength is slightly lower than the MOF blank water dispersion except for the chromate ions. This means that their existence has little contribution to the cumulative luminescence quenching. Nevertheless, the luminescence strength obviously reduces after adding the Cr_2_O_7_^2–^ ions (Figure 3(d)). This conforms that the complex **1** is a good material to the selectively detect the chromate ions in the water with a standard condition, and the existence of some other anions will not interfere with the selective detection.

**Figure 4.**
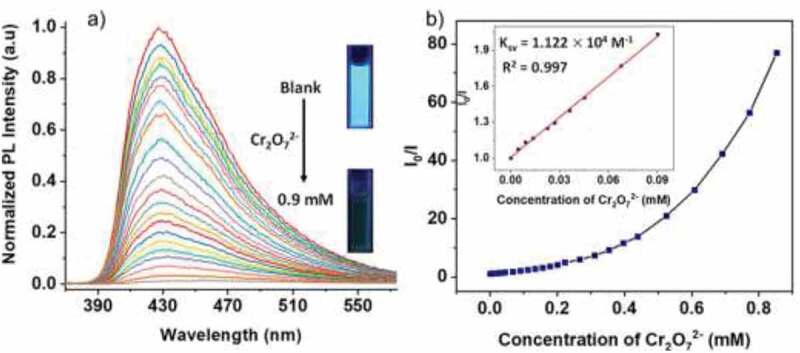
(a) Complex **1**’s photoluminescence spectra and (b) the diagram of Stern–Volmer plot increased gradually with the Cr_2_O_7_^2–^ ions concentration in the water. The linearity of quenching in a low range of concentration is reflected in the illustration

In order to determine the sensitivity of quenching reaction quantitatively, the experiments of fluorescence titration were conducted with diverse Cr_2_O_7_^2–^ ions concentrations. As the anion concentration of the analyte increased, the fluorescence spectrum was harvested (Figure 4(a)). When the Cr_2_O_7_^2–^ ions concentration up to 0.9 mM, the fully quenching was found. We calculated the constants of Stern–Volmer (*K*_SV_) via utilizing the curve of Stern–Volmer in a low range of concentration. The correlation coefficient of linear fitting is 0.997, which fits well with the curve. Nevertheless, the higher the concentration, the worse the linearity, indicating the existence of self-absorption or the process of energy-transfer. According to the curve of dose–response, the *K*_SV_ value of the Cr_2_O_7_^2–^ ion was calculated as 1.122 × 10^4^ M^–1^ (Figure 4(b)). The value of LOD (limit of detection) was calculated to be 1.04 μM by using the equation: LOD = 3σ/k, where σ stands for standard deviation, k stands for the slop of calibration curve of the luminescence intensity of compound upon addition of analyte (Table S1 and Fig S1). The lower values of LOD and higher values of *K*_sv_ display complex **1**’s outstanding ability of chromate ion sensing. In comparison with some other sensing materials, complex **1** can be used as an effective chromate ion sensing material in the medium of water.

### Compound significantly reduced the releasing levels of IL-18 and TNF-α

**Figure 5.**
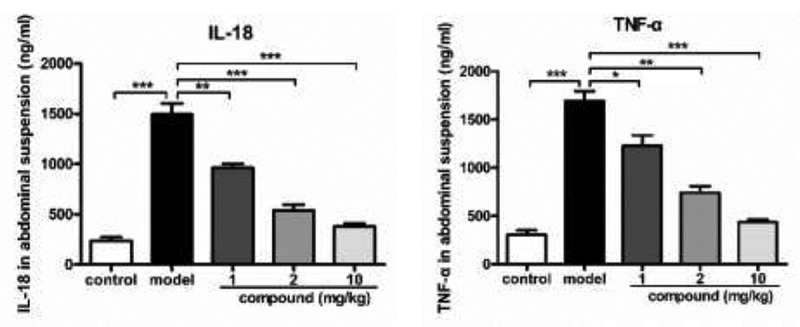
Significantly reduced releasing the TNF-α and IL-18 levels after conducting the compound treatment. We injected LPS into mice to construct the model or sepsis at 5 mg/kg concentration. The compound was applied to implement treatment at 1 mg/kg, 2 mg/kg and 10 mg/kg. The content of TNF-α and IL-18 in the abdominal suspension was determined via the ELISA test kit

After synthesizing the compound with novel structure, the treatment effect of compound against sepsis was evaluated. As during the sepsis procession, there was usually combined with an inflammatory factor storm, displayed as the enhanced TNF-α and IL-18 level, which could further cause the tissue damage. Thus, in this experiment, the ELIA was conducted and TNF-α and IL-18 level was determined. As the results showed in Figure 5, the levels of TNF-α and IL-18 in model mice of sepsis were significantly higher than the TNF-α and IL-18 level of the mice in the control group, indicating that the model group was more serious. After treated via the compound, the TNF-α and IL-18 level was reduced with the dose dependent pattern, indicating that compound possess excellent treatment effect against the sepsis.

### Compound obviously suppress the activation of the NF-κb signaling pathway

**Figure 6.**
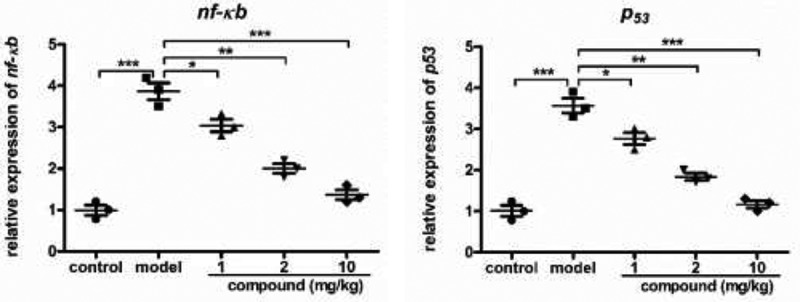
Obviously suppress signaling pathway of NF-κb activation levels after conducting the compound treatment. We injected LPS into mice to construct the model or sepsis at 5 mg/kg concentration. The compound was applied to implement treatment at 1 mg/kg, 2 mg/kg and 10 mg/kg. The signaling pathway of NF-κb activation levels in immune cells was determined with real time RT-PCR

In the experiment of ELISA, we have proved the inhibition of compound against the inflammatory cytokines releasing during sepsis. But the subordinate mechanism was still to be investigated. Thus, real time RT-PCR approach was in-depth performed and the signaling pathway of NF-κb activation levels in immune cells was measured. According to the results in the Figure 6, we find compared with control group, model group *p53* and *nf-κb* expression level was increased obviously. Under the exposure of compound, the *p53* and *nf-κb* expression level in the immune cells was obviously decreased with the dose dependent pattern.

## Conclusion

In conclusion, we have synthesized a metal-organic framework based on Zn impeller through utilizing 5-(benzimidazol-2-yl) isophthalic acid, a ployaromatic ligand. The compound **1** reflects a three-dimensional skeleton and an uncommon quadruple interactions of π–π between the two adjacent ligands of byia, and complex 1’s structure can be considered as the topology of ***nbo***. It is worth noting that the compound **1** has excellent water stability (which can be maintained in the water for at least a month). Most fascinating, in water, the compound **1** exhibits the strong blue luminescence, which can only be selectively quenched via the contaminant of the Cr_2_O_7_^2 –^ ion. The selective luminescence quenching with low limits of detection and High valueso of *K*_sv_ proved its better sensing property, which can be compared with the contemporary materials. The data of the ELIA detection strongly suggested that after treated through compound, the releasing of the TNF-α and IL-18 was suppressed. Next, the real time RT-PCR results displayed that the signaling pathway of NF-κb activation level was also decreased through implementing compound treatment. To sum up, the synthetic compound revealed outstanding treatment values for the sepsis, which could inhibit the inflammatory factor storm significantly.

## Data Availability

The data used to support the findings of this study are included within the article.
